# A Case of Hydramnios Complicating Twin Pregnancy

**Published:** 1885-09

**Authors:** A. H. Evans

**Affiliations:** Cason, Texas


### A CASE OF HYDRAMNIOS COMPLICATING TWIN PREGNANCY.

BY A. H. EVANS, M. D., CASON, TEXAS.

On the 24th day of April last I was callad to see Mrs H——, age 30; mother of three children. The woman had been suffering with cutting pains, as she expressed it, for two weeks, and was troubled with difficult respiration to such an extent that she could not stay in one position but a short time, except on her feet. She complained of feeling too large, compared with her other pregnancy. The physical *contour* of the abdomen was symmetrical, and measured 41½ inches at the umbilicus. The legs were swollen to the knees, and would pit on pressure. Detection of the pulsation of the fœtal heart was impossible. A vaginal examination was made, and the os found to be dilated to the diameter of a silver dollar. I waited two hours, and a second examination was made. The os was well dilated and soft. The amniotic sac was pushed into the vagina and ruptured the sac, and a small amount of water escaped. The right foot of a child was found presenting. The pains came on regularly, but the woman complained of inablilty to bear down; *all* muscular power seemed lost. I pushed up the right foot, and, grasping the left, brought both feet down. All went well until the shoulders engaged in the pelvic outlet, when I again had to assist nature by bringing down the shoulders. This I did by bringing the arms down. A large male child was now born, nearly in a putrid state. The cord was now tied and cut, and the abdomen not seeming to be any smaller, I examined, to find the head of a second child presenting with the bag of water drawn tightly over the head. I waited on the feeble uterine contraction for an hour and a half, then gave half a teaspoonful of fluid ex. ergot; waited twenty minutes, made an examination, and found amniotic sac distended into the vagina. It was ruptured, and a large amount of water escaped. The child's head was born, but no further progress of labor was made, notwithstanding uterine contractions were good. I at once proceeded to bring down the arms, but feeling the umbilical cord, wrapped around the child's neck three times, I believed it to be the cause of the non-progression of labor. Being satisfied that the child was dead, I cut the cord and disengaged it from around the neck of the child. The child was born as soon as the cord was cut. It nearly floated upon the bed, so great was the flow of water following it. This child, as I surmised, was dead, and, like the first, in a putrid state. It was a male, and much larger than the first. The amniotic fluid was the color of muddy coffee with sediments in it like coffee grounds. The quantity must have been more than a gallon, for it soaked through the bedding and ran off the bed on the floor. I waited fifteen minutes and tried to deliver the placenta by Crede's method, but failed. The womb would contract, but would not expel its contents. I gave half teaspoonful of fluid ext. ergot every hour for three hours, when two placentæ were expelled, the largest I ever saw. My patient made a rapid recovery, and is to-day able to do her usual amount of work.

The previous history of this case, as learned from the family, was that she had been treated two years ago for general dropsy, caused by malaria, and had never been in good health since.

